# Three-year clinical performance of direct restorations using low-shrinkage Giomer vs. nano-hybrid resin composite

**DOI:** 10.3389/fdmed.2024.1459473

**Published:** 2024-10-09

**Authors:** Tugba Toz-Akalin, Funda Öztürk-Bozkurt, Mahmut Kusdemir, Alev Özsoy, Emir Yüzbaşıoğlu, Mutlu Özcan

**Affiliations:** ^1^ School of Dentistry, Department of Restorative Dentistry, Istinye University, Istanbul, Türkiye; ^2^ School of Dentistry, Department of Restorative Dentistry, Istanbul Medipol University, Istanbul, Türkiye; ^3^ Department of Prosthodontics, School of Dentistry, Istanbul Galata University, Istanbul, Türkiye; ^4^ Center for Dental Medicine, Clinic of Masticatory Disorder of Dental Biomaterials, University of Zurich, Zurich, Switzerland

**Keywords:** clinical study, dental materials, Giomer, nano-hybrid resin composite, S-PRG filler, survival, multi-ions

## Abstract

**Objectives:**

The objective of this investigation was to compare the clinical performance of a nano-hybrid resin composite and a low-shrinkage Giomer resin composite.

**Material and methods:**

In total, 35 pairs of restorations were performed using either low-shrinkage Giomer (Beautifil II LS, Shofu Inc.) or nano-hybrid (Clearfil Majesty Posterior) resin composite in 35 patients by two operators using the relevant adhesives, i.e., FL-Bond II (Shofu Inc.) and Clearfil SE Bond (Kuraray), with the self-etching technique according to each manufacturer's instructions. Two clinicians assessed the restorations 2 weeks (baseline); 6 months; and 1, 2, and 3 years after the restorative procedures using FDI (World Dental Federation) criteria (Scores 1–5). Data were analyzed using the marginal homogeneity and McNemar tests. The survival rate was calculated using Kaplan–Meier survival analysis and the survival of the two groups was compared with the log-rank test (*p* = 0.05).

**Results:**

The mean observation period was 37.7 ± 6.8 months. All restorations completed their 3-year follow-up. The criteria were mainly rated with high (1 or 2) scores for quality in both groups. Only one restoration in the low-shrinkage Giomer resin composite group was accepted as a failure at the 2-year recall due to retention loss.

**Conclusion:**

At the 3-year follow-up, the performance of the restorations using the Giomer and the nano-hybrid resin composite were similar and clinically acceptable.

**Clinical relevance:**

The low-shrinkage Giomer resin composite exhibited a similar clinical performance to the nano-hybrid resin composite after 3 years in service with both materials displaying minor surface deteriorations at the 3-year recall.

**Clinical Trial Registration:**

https://clinicaltrials.gov, identifier: NCT02823769.

## Introduction

Resin composite are regarded as the preferred direct restorative material applied by dental clinicians due to their improved esthetic and functional properties as well as the increasing esthetic expectations of the patients (1). The literature shows that resin composite can demonstrate long-term clinical success in restorations of posterior teeth. In a review that included such restorations, the success rate of direct resin composite restorations applied in the posterior region was found to be 48% for up to 33 years. The main causes of failure identified were fractures and secondary caries (2). The idea that polymerization shrinkage is a cause of this failure has led researchers to minimize this shrinkage by improving the filler content of resin composite or modifying adhesive systems and techniques. In addition, resin composite with anti-caries properties have become another research aim (3). Current developments in restorative dentistry have begun to focus on the ability of restorative materials to exhibit bioactive properties. Although the term bioactive is not very current, it is still a controversial issue. However, since ion release is one of the necessities for a material to be considered bioactive, today's restorative dentistry has started to focus on ion-releasing restorative materials (1). Glass ionomers are accepted as the first group of dental restorative materials able to fulfill some of the properties expected of bioactive restorative materials. The release of fluoride from glass ionomers is responsible for promoting the biomineralization of mineral-depleted hydroxyapatite and forms an acid-resistant layer in which hydroxyapatite is converted into fluorine-apatite (4, 5). Nevertheless, the clinical applicability of glass ionomers is constrained to low-stress bearing areas on account of their inadequate flexural strength, resilience, and resistance to wear, as evidenced in the literature (5).

The idea of adapting the ion release properties to resin-based materials led researchers to develop pre-reacted glass (PRG) technology, and these materials were named Giomers (6). In PRG technology, in the presence of water, an acid–base reaction occurs between fluoride-containing glass particles and polymer-containing acid, resulting in a glass-ionomer phase before dispersal into the resin (7, 8). The pre-reacted glass-ionomer phase surrounding the glass core allows the S-PRG (surface pre-reacted glass ionomer) filler to release fluoride ions (F−).

In addition to the fluoride ions, five different ions, namely strontium (Sr2+), borate (BO33−), sodium (Na+), silicate (SiO32−), and aluminum (Al3+) ions, are released from this material due to the specially fabricated fluoro-boro-aluminosilicate glass core (7). The basis of a Giomer is the S-PRG filler and it has started to be used and tested as a bioactive agent in different dental products apart from resin composite such as resin cements, fissure sealants, coating resin materials, temporary fillings, and polishing pastes. Many studies have demonstrated that Giomers exhibit antibacterial effects against oral bacteria (9, 10). Therefore, the success of Giomer resin composite with anti-cariogenic properties should be clinically discussed.

In addition to PRG fillers, Giomer resin composite contain conventional silanated micro and macro fillers. In relation to these contents, Giomer resin composite can be considered restorative materials with similar success as conventional resin composite in terms of high fracture toughness and flexural strength (5, 11). Beautifil (Shofu, Kyoto, Japan) was developed using S-PRG technology and was indicated for Class I to Class V cavities. Developments in PRG technology have led to the emergence of the modified S-PRG filler, which includes a three-layer texture with a glass core of multifunctional fluoro-boro-aluminosilicate glass and bifacial layers forming the PRG phase around the glass core, and a reinforced modified layer covering the surface of this PRG phase. Beautifil II, developed with this novel technology, is considered a second-generation Giomer (12). The Flowable Giomer resin composite, which offers applications in a different viscosity, and low-shrinkage Giomer resin composite are other current Giomer technologies (3). Low-shrinkage Beautifil II is a current resin composite and an example of the latest second-generation PRG technology, showing a bioactive effect with six types of multi-ion released from the S-PRG filler (6).

Although not widely mentioned in the literature, Giomer resin composite have been shown to be suitable definitive restoratives in clinical studies (13, 14). The study of Gordan et al. (14), with the longest observation period in the literature of 13 years, showed that these restorations have an acceptable clinical prognosis. However, in this study, only a clinical follow-up of the material was conducted without a comparison group with a conventional composite resin that could be used as a control (15). There are not many studies in the literature that have compared Giomer resin composite with conventional ones (16, 17) or glass ionomers (18, 19) in terms of clinical success, and these studies are generally designed for Class V restorations. Randomized clinical trials are necessary to compare and assess the clinical success of Giomer resin composite with other restorative materials. Therefore, the aim of this controlled clinical study was to evaluate and compare the clinical behavior of a low-shrinkage Giomer resin composite (LSG) belonging to second-generation PRG technology and a nano-hybrid (HC) resin composite for Class I and II type cavities. The previous results of this study revealed that the performance of the LSG and HC restorations was similar and clinically acceptable (20). The null hypothesis of this study was that there would be no difference in the clinical prognosis between low-shrinkage Giomer resin composite and nano-hybrid resin composite restorations.

## Materials and methods

### Study design

**Figure 1:**
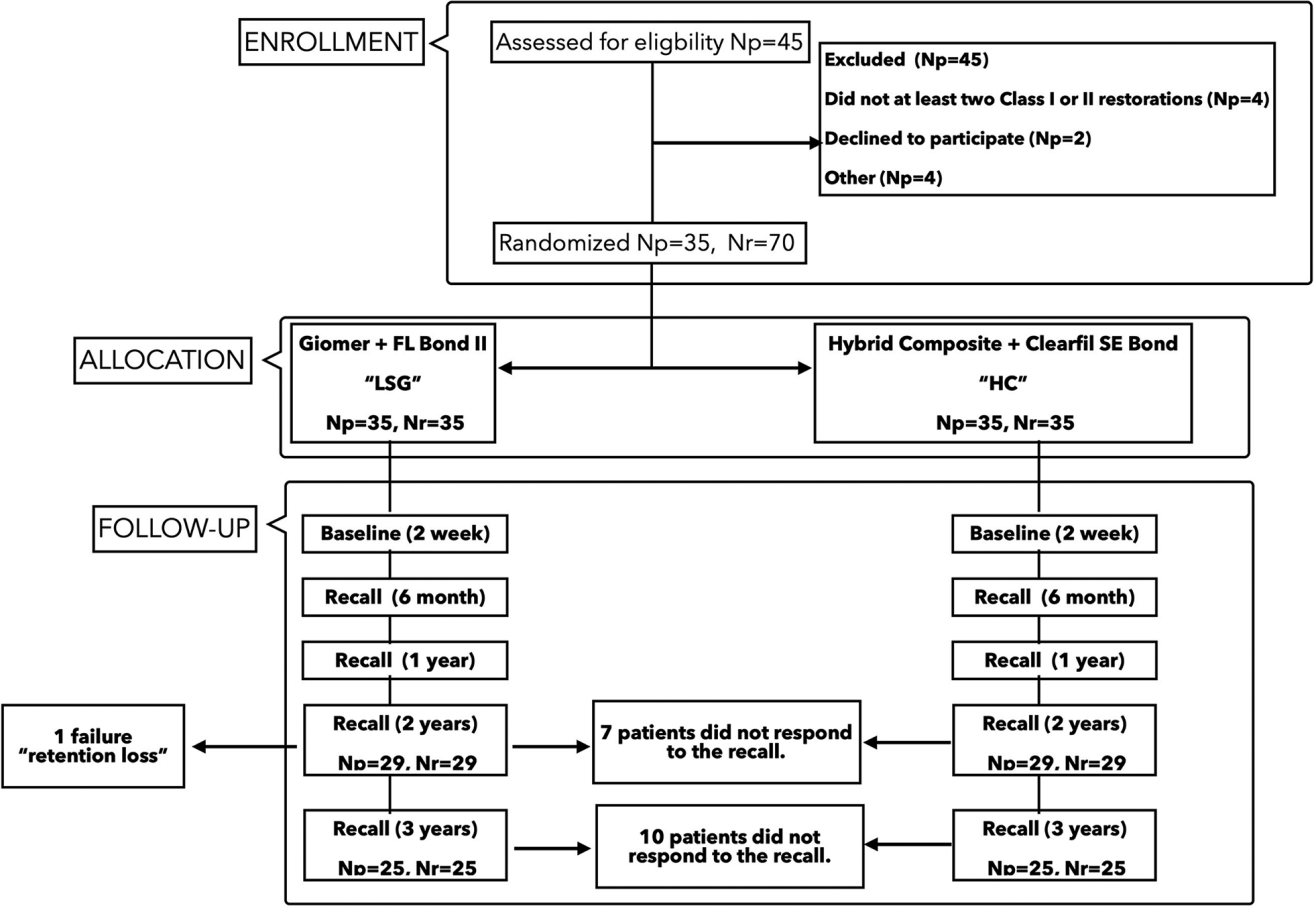
Flowchart of the study. Np, number of patients; Nr, number of restorations.

The study design of this clinical research is detailed in the article comparing the 2-year clinical behaviors of the materials tested in the present research (20) and depicted in a flow chart (Figure 1). The requisite sample size was calculated using the G-Power statistical software (version 3.1.9.4). In consideration of the relatively modest standardized effect size (Cohen's d = 0.5) with 80% power and an alpha error of 5%, the requisite sample size was determined to be 70 restorations. In total, 35 patients, aged between 18 and 47 years, with good to moderate oral hygiene and with at least two primary caries, were referred to Istanbul Medipol University, Dental School, Department of Restorative Dentistry to receive, at random, 35 pairs of fillings. Each pair of teeth was randomly assigned to receive either a low-shrinkage Giomer resin composite (Beautifil II LS) or a nano-hybrid resin composite (Clearfil Majesty Posterior, Kuraray, Osaka, Japan). The restorations were applied by two operators with postgraduate experience in restorative dentistry. A total of 70 teeth (comprising 35 molars and 35 premolars) were prepared and restored with either the Giomer (*n* = 35) or nano-hybrid resin composite (*n* = 35) in a randomized manner, with each patient serving as their own control. Table 1 contains information about the chemical content of the materials used in the research, the manufacturers, and their brands. The patients included in the study according to the inclusion and exclusion criteria in Table 2 were informed about the research details and enrollment in the recall program and their signed consent was obtained after receiving approval from the ethical committee of the university (Vote number of the local Ethical Committee no:10840098-604.01.01-E.3215; Clinical Trial Registration Number: NCT02823769).

**Table 1:** Brand, type, manufacturer, and chemical composition of the main materials used in this study.

Brand	Type	Manufacturer	Chemical composition
Beautifil II LS	Low-shrinkage Giomer resin composite	Shofu Inc., Kyoto, Japan	Multifunctional glass and S-PRG filler based on fluoro-boro-aluminosilicate glass, pre-polymerized filler, nano filler, photoinitiator, low-shrinkage urethane diacrylate, bis-MPEPP, bis-GMA, TEGDMA
Clearfil Majesty Posterior	Nano-hybrid resin composite	Kuraray Medical, Tokyo, Japan	bis-GMA, TEGDMA, hydrophobic aromatic dimethacrylate, glass ceramics, surface treated alumina micro-filler, silica filler
FL-Bond II	Self-etching two-step Giomer adhesive system	Shofu Inc.	Primer: Water, ethanol, carboxylic acid monomer, phosphoric acid monomer and initiator
			Adhesive: S-PRG filler based on fluoro-boro-aluminosilicate glass, UDMA, TEGDMA, 2-HEMA, initiator
Clearfil SE Bond	Two-step self-etch adhesive system	Kuraray Medical	Primer: 10-MDP, HEMA, hydrophilic dimethacrylate, di-camphorquinone, aromatic tertiary amine, water
			Adhesive: 10-MDP, bis-GMA, HEMA, hydrophilic dimethacrylate, photoinitiator, aromatic tertiary amine, silanized colloidal silica

S-PRG, surface pre-reacted glass ionomer; bis-MPEPP, bisphenol A polyethoxy dimethacrylate; bis-GMA, bisphenol A-glycidyl methacrylate; TEGDMA, triethylene glycol dimethacrylate; UDMA, urethane-dimethacrylate; 2-HEMA, 2-hydroxyethyl methacrylate; 10-MDP, 10-methacryloyloxydecyl dihydrogen phosphate.

**Table 2:** The list of inclusion and exclusion criteria considered for the enrollment of patients in this study.

Inclusion criteria	Exclusion criteria
• Primary carious lesions: Class I and II restorations • No obvious untreated caries or dental health problems (gingival and periodontal health problems; mucosa pathologies; erosive tooth wear, attrition, and abrasion, developmental defects; and dental trauma, regularly checked by a dentist) • Good or moderate oral hygiene (“O'Leary Plaque Score Index” plaque score of less than 30% in the anterior region before treatment) • No untreated periodontal disease (only DPSI 1 or 2) • Subjects over the age of 18, classified according to the ASA as ASA I or II, with good oral hygiene, and free of periodontal disease (probing depth and attachment levels within normal limits, no furcation involvement, and no mobility) • Subjects had to agree to keep the scheduled recall appointments for data collection and maintenance and planned to stay in the geographic location for at least 3 years	• Composite or amalgam removal • Caries extends to cementoenamel junction in Class II • Considerable horizontal or vertical mobility of teeth: tooth mobility index score 2 or 3 • Considerable periodontal disease without treatment (DPSI 3−, 3+, and 4) • Endodontic treatment with extensive loss of tooth tissues • Patients who still want to bleach their teeth or had bleached their teeth less than 3 weeks ago • Excluding the teeth, without opposing natural dentition (either intact or restored with intracoronal or extracoronal fixed restorations), and a minimum of 20 teeth • Subjects who presented with severe wear facets or reported parafunctional activities such as clenching or nocturnal bruxism • Subjects who had been restored with a removable partial dental prosthesis (RPDP), unless the RPDP replaced the tooth that was planned to be restored in the study • Subjects who were pregnant during the duration of the study • Subjects who were known to be allergic to the ingredients of resin materials

### Treatment protocol

In total, 35 patients (17 women and 18 men,) with a mean age of 29 ± 9 years (between 18 and 47 years) received 35 pairs of restorations between February 2016 and May 2017. The individuals included in the study had good to moderate oral hygiene with at least two primary carious lesions. The primary carious lesions scheduled for restoration were identified as Code 3 and Code 4 according to the ICDAS clinical visual system. Each pair of restorations were performed with either LSG (Beautifil II LS, “A1 shade”) or HC (Clearfil Majesty Posterior, Kuraray, Osaka, Japan, “A1 shade”) using randomization software (www. randomizer.org) by two operators (both with more than 12 years of experience). Prior to the restorative procedures, the operators performed cold pulp tests by spraying ice crystals (Nexcare, 3M ESPE, St Paul, MN, USA) onto a cotton pellet and applying it to the buccal surfaces of the teeth. All the cavity walls were finished with extra-fine burs (pin diamond finishing burs, Frank Dental GmbH, Gmund, Germany) along the preparation boundaries. All the cases were photographed before the restorative procedures, after the preparation, and at the end of the restoration. The operative field was washed with an air/water spray, gently air dried, and then carefully isolated with suction and cotton rolls. A metal matrix band and wooden wedge were used in the class II cavities during the restorative procedures (Adapt SuperCap Matrices, KerrHawe, Bioggio, Switzerland). In the Giomer group, self-etch adhesive (FL-Bond II, Shofu Inc.) was applied using the self-etching technique and photopolymerized for 10 s (Bluephase Style, Ivoclar Vivadent, Schaan, Liechtenstein). The power output of the unit was 1,100 mW/cm^2^ and was measured with a radiometer (Cure-Rite, Dentsply Caulk, Ontario, Canada) before application. In the nano-hybrid resin composite group, a different self-etch adhesive (Clearfil SE Bond, Kuraray) was applied with a similar self-etching technique. Both resin composite were applied to the cavities using an incremental oblique technique (max 2 mm). Each increment was photopolymerized for 40 s. After the polymerization of the restorations, early occlusal contacts were checked and removed. Furthermore, any interproximal contacts were checked with dental floss, and the necessary corrections were made. The finishing and polishing procedures of the restorations were performed using discs and paste (Super-Snap and DirectDia Paste, Shofu Inc.) and rubber tips (Compo Master, Shofu Inc.) according to the manufacturer’s instructions (20). In total, 70 teeth were randomly prepared and restored in each patient with either LSG (*n* = 35) or HC (*n* = 35) resin composite.

### Clinical assessment

Two different independent clinicians with more than 15–20 years of clinical experience evaluated the restorations according to the details that have been mentioned in a previous article (20). The e-calib system (www.e-calib.info) was preferred for the calibration between observers in this research and a minimum of 80% intra-agreement was accepted (15, 21). The evaluations were initially performed 2 weeks after the restorative procedures (baseline) and then annually according to FDI (World Dental Federation) criteria for 3 years. For some parameters, a semi-quantitative clinical evaluation (SQUACE) was carried out by the two independent observers. A SQUACE is a method for easily measuring marginal deteriorations. On an evaluation sheet with sketches of the occlusal and mesio- and disto-proximal parts of the Class I and II restorations, the extent of the observed condition was outlined using different colored pencils according to the specified criteria. The lines were then related to the size of the sketch and scored according to defined categories (22). The patients were requested to call in the case of any complaints about the performed restorations.

### Statistical analysis

Comparisons of data which were expressed by number and percentages were performed using the marginal homogeneity and McNemar tests. The survival rate was calculated using Kaplan–Meier survival analysis and the survival of the two groups was compared with the log-rank test. In all tests, the predetermined type 1 error rate (alpha) was accepted as 0.05 (SPSS Statistics, v25.0, IBM, NY, USA).

## Results

**Figure 2:**
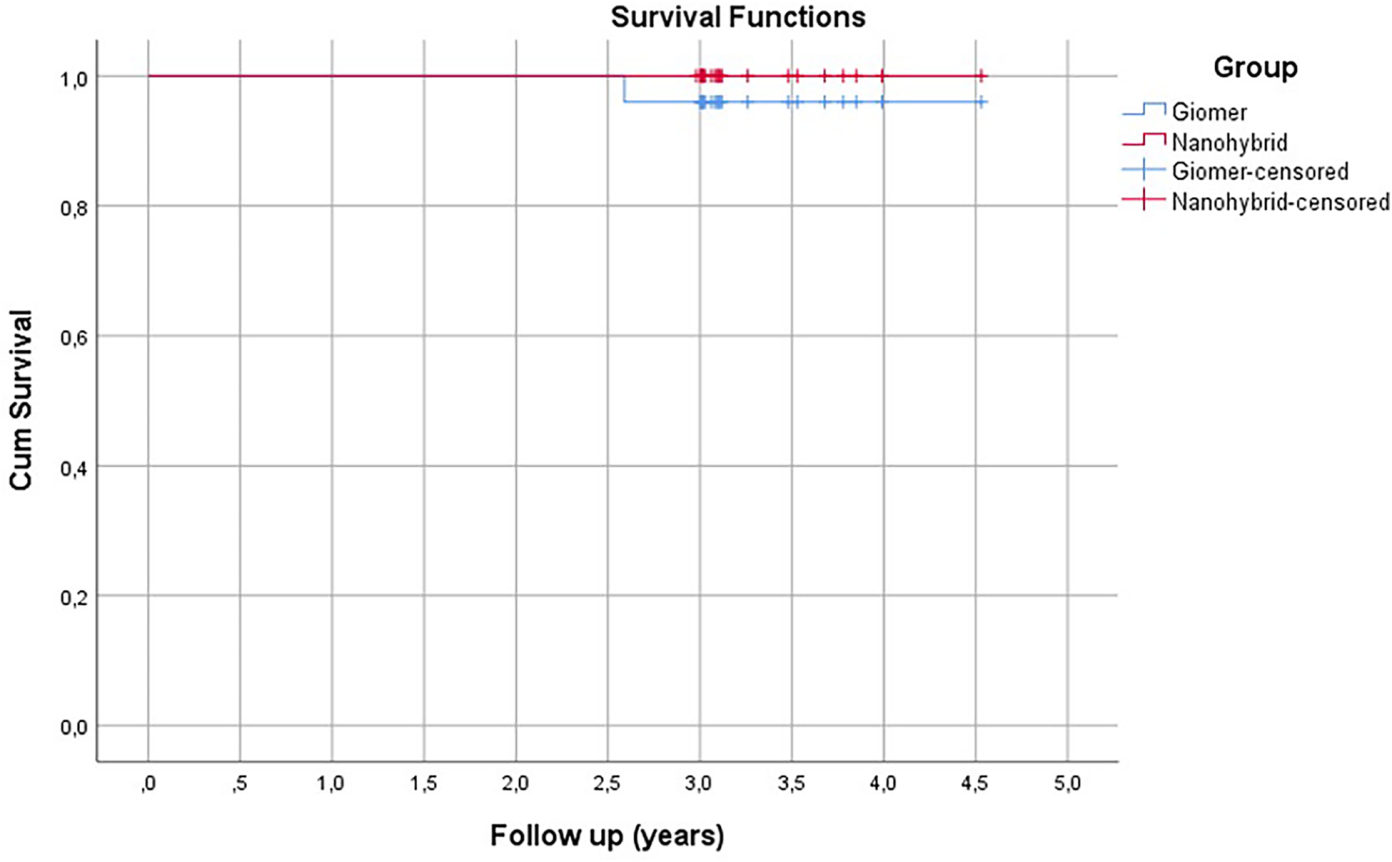
Event-free survival rates of resin composite restorations for Class I and II cavities (*n* = 25). Mean survival time in the Giomer group was 54.17 ± 0.93 months (95% CI = 52.36 ± 55.98).

The distribution of the 70 restored teeth (47 premolars and 23 molars) regarding location, tooth type, and restoration type for the two restorative materials tested are given in Table 3. No cracks were observed after cavity preparation in any tooth tissues. In total, 50 restorations (71%) were observed at the 3-year recall with a mean observation period of 37.7 ± 6.8 months (min: 35.4, max: 44.2 months). In the 3-year evaluation, 10 patients with 20 restorations did not attend the scheduled recalls for various reasons, including relocation to a different city or change of telephone number. During the 3-year observation period, no contact point loss was observed in any of the restorations that required repair. No teeth fractures or endodontic failures were observed during the 3-year clinical follow-ups. One restoration from the LSG group showed retention loss at the 2-year follow-up and was accepted as a failure (Tables 4–6). The overall survival rate of the LSG group was 96% and, for the HC group, it was 100% (Kaplan–Meier, Figure 2). When the survival of the two groups was compared with the log-rank test, no statistically significant difference was observed between the two tested groups (*p* = 0.317).

**Table 3:** Distribution of the 70 restored teeth regarding location, tooth type, restoration type in the maxilla and mandible for the two restorative materials tested, a low-shrinkage Giomer resin composite (LSG) and a conventional nano-hybrid resin composite (HC).

Location	Teeth	One-surface		Two-surface		Three-surface	
		LSG	HC	LSG	HC	LSG	HC
Maxilla	Premolar	—	—	14	17	4	1
	Molar	1	—	5	6	—	—
Mandible	Premolar	—	—	4	6	—	—
	Molar	2	3	4	2	1	—
Total		3	3	27	31	5	1

**Table 4:** Number and percentage of scores for the esthetic properties of LSG and HC according to FDI criteria 1, 2a, and 3.

Esthetic properties						
Score	Surface luster *n* (%)		Surface staining *n* (%)		Color stability and translucency *n* (%)	
	Third year (*n* = 25)		Third year (*n* = 25)		Third year (*n* = 25)	
	LSG	HC	LSG	HC	LSG	HC
1	0 (0)	0 (0)	19 (76)	19 (76)	0 (0)	0 (0)
2	25 (100)	25 (100)	5 (20)	5 (20)	24 (96)	23 (92)
3	0 (0)	0 (0)	1 (4)	1 (4)	1 (4)	2 (8)
4	0 (0)	0 (0)	0 (0)	0 (0)	0 (0)	0 (0)
5	0 (0)	0 (0)	0 (0)	0 (0)	0 (0)	0 (0)

Number and percentage of scores for esthetic properties of the low-shrinkage Giomer resin composite (LSG) and the conventional nano-hybrid resin composite (HC) according to the FDI criteria.

1: Clinically excellent/very good; 2: Clinically good (very good after polishing); 3: Clinically sufficient/satisfactory (minor shortcomings, no unacceptable effects but not adjustable w/o damage to the tooth); 4: Clinically unsatisfactory (but reparable); 5: Clinically poor (replacement necessary).

**Table 5:** Number and percentage of scores for the functional properties of LSG and HC according to FDI criteria 6b, 8, and 7–10.

Functional properties						
Score	Fractures and retention *n* (%)		Contact point *n* (%)		Wear, Patient's view *n* (%)	
	Third year (*n* = 25)		Third year (*n* = 25)		Third year (*n* = 25)	
	LSG	HC	LSG	HC	LSG	HC
1	20 (80)	22 (88)	23 (92)	25 (100)	25 (100)	25 (100)
2	4 (16)	3 (12)	2 (8)	0 (0)	0 (0)	0 (0)
3	1 (4)	0 (0)	0 (0)	0 (0)	0 (0)	0 (0)
4	0 (0)	0 (0)	0 (0)	0 (0)	0 (0)	0 (0)
5	0 (0)	0 (0)	0 (0)	0 (0)	0 (0)	0 (0)

Number and percentage of scores for the functional properties of the low-shrinkage Giomer resin composite (LSG) and the conventional nano-hybrid resin composite (HC) according to the FDI criteria.

1: Clinically excellent/very good; 2: Clinically good (very good after polishing); 3: Clinically sufficient/satisfactory (minor shortcomings, no unacceptable effects but not adjustable w/o damage to the tooth); 4: Clinically unsatisfactory (but reparable); 5: Clinically poor (replacement necessary). All scores for the marginal adaptation and wear, patient's view criteria are displayed as 1 and are stated in the same column.

**Table 6:** Number and percentage of scores for the biological properties of LSG and HC according to FDI criteria 11, 12, and 13.

Biological properties						
Score	Postoperative hypersensitivity and tooth vitality *n* (%)		Recurrence of caries, erosion, and abfraction *n* (%)		Tooth integrity (enamel cracks) *n* (%)	
	Third year (*n* = 25)		Third year (*n* = 25)		Third year (*n* = 25)	
	LSG	HC	LSG	HC	LSG	HC
1	25 (100)	25 (100)	24 (96)	24 (96)	24 (96)	24 (96)
2	0 (0)	0 (0)	1 (4)	1 (4)	1 (4)	1 (4)
3	0 (0)	0 (0)	0 (0)	0 (0)	0 (0)	0 (0)
4	0 (0)	0 (0)	0 (0)	0 (0)	0 (0)	0 (0)
5	0 (0)	0 (0)	0 (0)	0 (0)	0 (0)	0 (0)

Number and percentage of scores for the biological properties of the low-shrinkage Giomer resin composite (LSG) and the conventional nano-hybrid resin composite (HC) according to the FDI criteria.

1: Clinically excellent/very good; 2: Clinically good (very good after correction); 3: Clinically sufficient/satisfactory (minor shortcomings with no adverse effects but not adjustable without damage to the tooth); 4: Clinically unsatisfactory (repair for prophylactic reasons); 5: Clinically poor (replacement necessary).

According to the esthetic parameters, the marginal quality scores (SQUACE) were usually scored as 1 during the 3-year follow-up. For the surface staining criteria, 12 restorations (6 from the LSG group and 6 from the HC group) received a score of 2, and 1 restoration from each group scored a 3. As for the surface luster scores, one restoration in each group received a score of 1 and other restorations received a score of 2 because they were observed to have slight pores on the restoration surfaces. According to the 3-year data, there were no statistically significant differences between the two materials in terms of esthetic parameters (*p* = 1.000).

According to the functional parameters, occlusal wear of the restorations was observed to be similar to the enamel wear at the 3-year follow-ups, while color match and translucency showed minor deviations across all the resin restorations (Score 2). Two restorations from the HC group were given a score of 2 (slightly deficient contour) for the approximal anatomical form criteria, but they did not require any intervention. When the functional parameters were evaluated, it was determined that there was no statistically significant difference between the two groups for all the criteria (*p* = 1.000).

**Figure 3:**
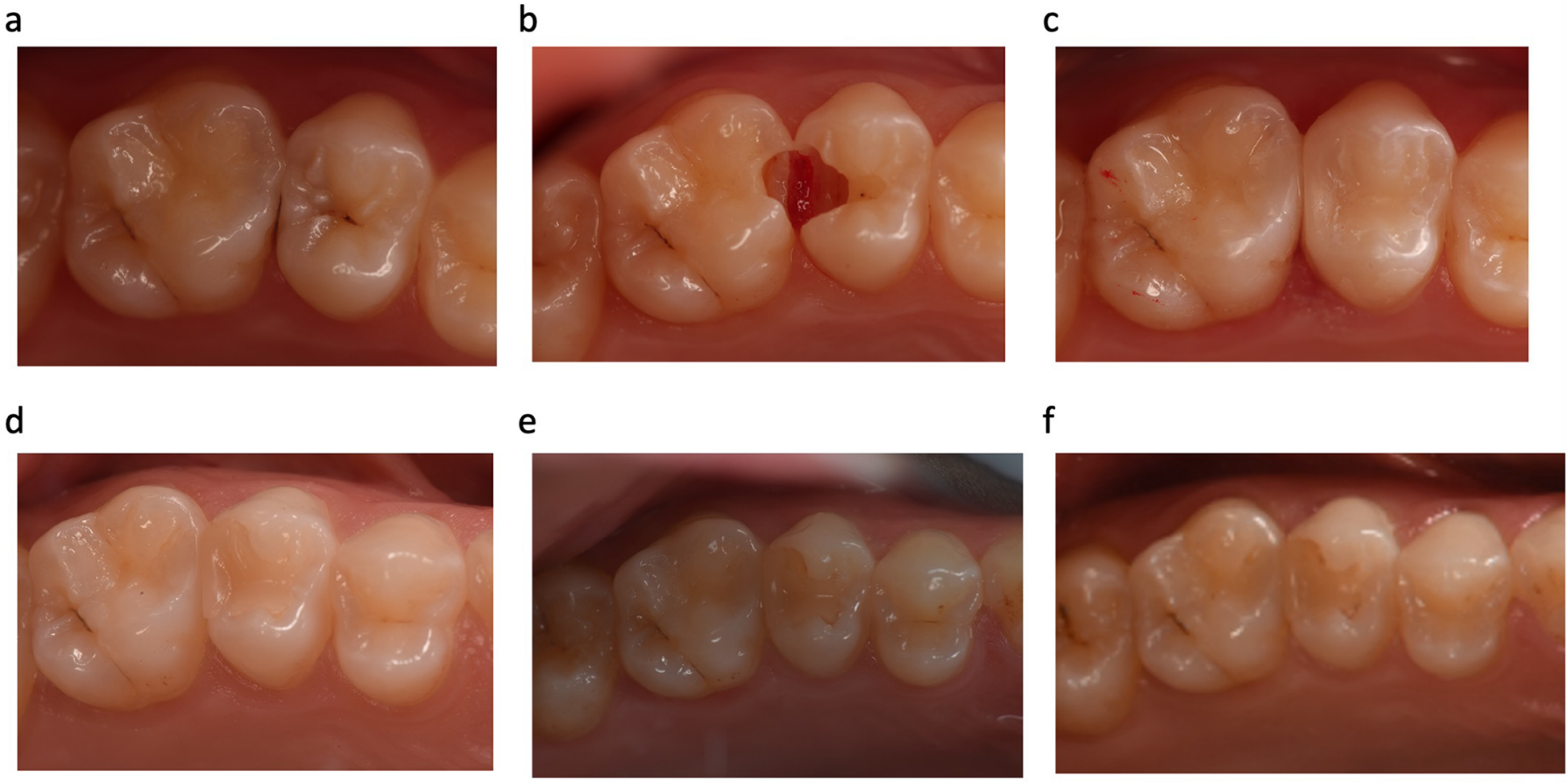
Representative photographs of **(a)** before cavity preparation (tooth no. 15 = low-shrinkage Giomer resin composite and tooth no. 16 = nano-hybrid resin composite); **(b)** after cavity preparation; **(c)** 2 weeks after filling placement (baseline); **(d)** and at the 1-, **(e)** 2-, and **(f)** 3-year follow-ups.

**Figure 4:**
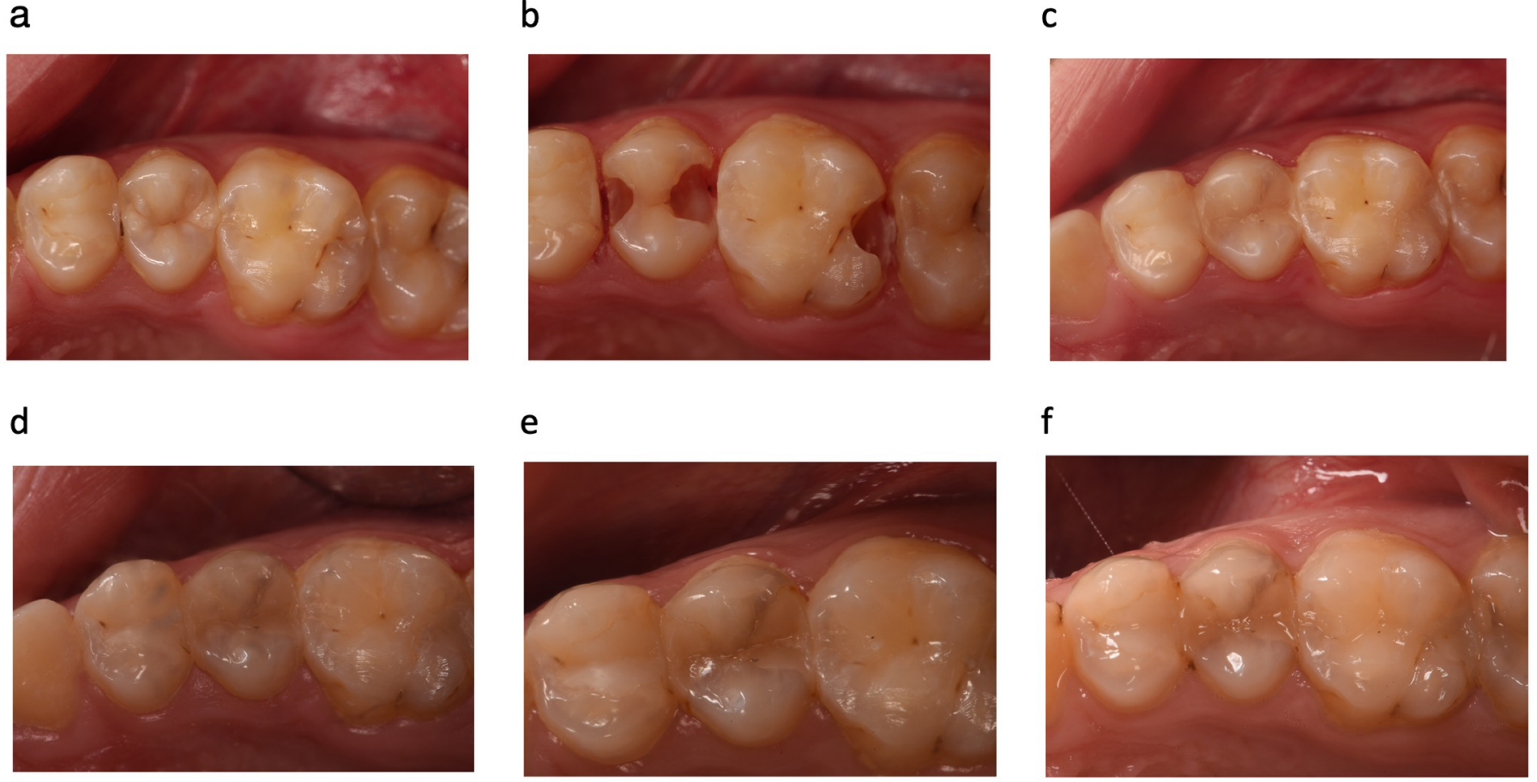
Representative photographs of **(a)** before cavity preparation (tooth no. 25 = low-shrinkage Giomer resin composite and tooth no. 26 = nano-hybrid resin composite); **(b)** after cavity preparation; **(c)** 2 weeks after filling placement (baseline); **(d)** and at the 6-month **(e)** and 2- **(f)** and 3-year follow-ups. Marginal staining was observed in the LSG group after the 2-year follow-up (tooth no. 25, scored as 3).

Regarding to the biological parameters, patient opinion (postoperative hypersensitivity was not observed) generally received a score of 1 after 3 years. A small secondary carious lesion (detected from the radiograph) in the HC group was observed after the 1-year recall which did not require repair or replacement. It was further evaluated at the 2-year follow-up and no progress was observed. This restoration could not be evaluated during the 3-year control period since the patient had moved out of the city. When the biological parameters were considered, the two groups did not show a statistically significant difference for any criterion (*p* = 1.000). In summary, there were no significant differences observed between the two restorative materials (*p* > 0.05) after 3 years. Figures 3A–F and Figures 4A–F show representative photos of the restorations using both resin composite.

## Discussion

The present clinical study aimed to compare two resin composite with different properties, one of which was a Giomer resin composite and the other was a nano-hybrid resin composite. The null hypothesis of this study was that there was no difference in the clinical performance of each resin composite. The clinical success of Giomer resin composite has been tested in clinical studies comparing these materials with glass ionomers, resin composite, and among themselves. In a clinical study comparing Giomers with glass ionomers, it was concluded that Giomer resin composite exhibited superior surface finishing and marginal adaptation, however, they showed similar retention compared to resin-modified glass ionomers (18). This smooth surface features of these materials was explained by this being typical of resin composite. In line with this explanation, the Giomer resin composite restorations exhibited similar clinical behavior to those in the nano-hybrid resin composite group in terms of surface properties in the present study.

In the clinical research literature on Giomer resin composite, a 3-year observational study comparing the traditional and flowable forms of these materials in Class V cavities emphasized that they provide similar clinical success (23). In a different study comparing the clinical prognoses of these two materials in conservative Class I cavities, restorations conducted using conventional form Giomer resin composite achieved clinically better results compared to the flowable form after 3 years in service (24). The longest follow-up period in a study in the literature on Giomer resin composite was 13 years, after which most of the restorations maintained acceptable clinical results (14). In this long-term study, more than half of the restorations evaluated were found to be clinically successful. In half of these clinically acceptable restorations, slight changes were observed in criteria such as color match, marginal adaptation, and marginal staining (14). In the present study, a loss of retention was observed in the Giomer resin composite group after 2 years of clinical service. Technical sensitivities encountered during the application of adhesive systems and different factors related to the application technique of the operators and the adhesive systems can be attributed to this failure. In addition to these reasons, providing isolation with cotton rolls rather than a rubber dam can also be considered a limitation in this clinical study.

Clinical research comparing conventional and Giomer resin composite in the literature has shown similar results with the present study (25). Considering the clinical literature involving Giomer resin composite, it has been observed that these materials have similar postoperative sensitivity and secondary caries findings as the restorative materials they are compared to (19, 26). In the present study, no postoperative sensitivity was observed at any follow-up period. Giomer resin composite have scored worse than other restorative materials for retention (19) and marginal staining (16) criteria in some studies. For the surface luster parameter evaluated in the present study, slightly dull surfaces were generally observed for both tested materials. This surface deterioration could be associated with factors such as the finishing and polishing techniques or the operator rather than the properties of the materials. A systematic review of the literature to evaluate whether the final surface roughness of anterior composite restorations is affected by the interaction between the resin composite and polishing systems supports this interpretation. The findings indicated that there is an absence of sufficient evidence to determine whether the combination of composite and polisher influences surface roughness (27).

Considering the functional parameters in this study, retention loss was determined to have occurred after 2 years in a restoration belonging to the Giomer resin composite group. Durable adhesion to tooth tissues is one of the most important prerequisites that could directly affect the clinical success of adhesive restorative treatments (28). A clinical study of direct adhesive posterior restorations indicated that the marginal deterioration incidence was mostly due to the adhesive system used in the restorative procedures (29). Despite all the developments in adhesive dentistry, no novel adhesive strategy is exempt from technique sensitivity stemming from the adhesion procedures, and significant concerns have been detailed in the literature about inter facial aging due to degredation of the adhesive interface (28). In short-term clinical studies in which self-etch adhesives have been used in the application of direct adhesive posterior restorations, it has been stated that marginal integrity is generally the most adversely affected parameter (30, 31). In this study, two-stage self-etch adhesive systems were preferred in the restorative procedures. The adhesive system applied in the nano-hybrid resin composite group was Clearfil SE Bond, which contains 10-methacryloxydecyl dihydrogen phosphate (10-MDP) as the monomer content. It has been reported that two-step self-etch adhesive systems containing 10-MDP generally have similar adhesive efficiency as that of three-step etch-and-rinse adhesive systems (28, 32). Apart from the applied adhesive system, the preferred adhesive technique is also important for the clinical prognosis of the resin composite restorations. In a study in which the application of Clearfil SE Bond with either the self-etch or selective etching adhesive technique was clinically compared, more marginal defects at the enamel side were found in the self-etch technique group (33). Similar to Clearfil SE Bond, the adhesive system applied with the Giomer resin composite, FL-Bond II, displays mild acidity with a pH of 2.4. In an *in vitro* study, it was reported that the application of FL-Bond II using the selective etching technique increased the adhesive bond strength (34). In this study, apart from the loss of retention observed in one case in the Giomer resin composite group, no difference was observed in terms of the clinical success of the materials. After 3 years of clinical observation, the success of both materials was found to be acceptable; however, that the effect of the applied adhesive technique on the clinical behavior of these restorative materials may reveal different results in the long term should not be ignored.

Apart from 10-MDP, another monomer frequently observed in the structure of adhesives is hydroxyethyl methacrylate (HEMA). In this study, both the adhesive systems tested contained HEMA and the two materials exhibited similar clinical findings after 3 years of evaluation. The loss of retention observed only in the Giomer group can be described as debonding and caused by failure of adhesion rather than swelling of the restorative material. Considering this situation, the retention loss could be attributed to the resin content of the adhesive system. Studies have emphasized that HEMA increases the water uptake of the adhesives and causes gradual hydrolytic degradation (16, 35).

It has been reported in dental literature that matrix metalloproteinases (MMPs), as a family of enzymes with the function of degrading the extracellular matrix, adversely affect dental adhesion over time (9, 36, 37). Therefore, studies in adhesive dentistry have been focused on enzyme inhibition (38) or biomimetic remineralization strategies (39) to slow down the degradation, to increase the rate of successful adhesion in the long term. In addition to the remineralization effect of PRG technology (40, 41), due to its S-PRG content, this resin composite inhibits dentin matrix degradation by acting similarly to 2% chlorhexidine digluconate, a known MMP inhibitor (36, 37). However, the mildly acidic self-etch adhesive system Clearfil SE Bond used in the nano-hybrid resin composite group has been found to cause a lower rate of bond degradation than other adhesive techniques in the literature (42, 43). Given that this study aimed to examine the bioactive effect of S-PRG and the long term clinical evaluation of its effects on MMPs, choosing an adhesive system that did not show a similar effect with a comparison group can be considered as one of the limitations of this study. However, long-term clinical follow-up is required to clearly reveal the possible effects of Giomer resin composite on MMP activity and, consequently, on adhesion success.

The exchange mechanism in the glass ionomer phase gives the S-PRG filler the ability to release and recharge fluoride ions, which can be described as the most effective agent against caries. Therefore, Giomer resin composite have been reported to release high concentrations of fluoride ions and can be recharged when exposed to a 5,000 ppm sodium fluoride solution for 5 min (9, 44–46). Considering that the most likely cause of failure in resin composite restorations is secondary caries (2), it can be thought that this release mechanism may provide an advantage to Giomer resin composite in long-term clinical evaluation in this respect. For this reason, long-term clinical evaluations are needed to demonstrate the success of Giomer resin composite.

## Conclusions

After 3 years in use, Giomer resin composite showed similar clinical success in Class I and II cavities compared to the nano-hybrid resin composite tested.

## Data Availability

The datasets presented in this article are not readily available. Requests to access the datasets should be directed to mkusdemir@medipol.edu.tr.
